# A novel portable *in situ* printer for hydrogel multi-structure molding and cell printing

**DOI:** 10.1063/5.0176301

**Published:** 2023-12-06

**Authors:** Huazhen Liu, Yi Zhang, Zhian Jian, Chuang Gao, Chunxiang Lu, Qiqi Dai, Hao Qiao, Yuanyuan Liu

**Affiliations:** 1 School of Medicine, Shanghai University, Shanghai 200444, People's Republic of China; 2 School of Mechatronic Engineering and Automation, Shanghai University, Shanghai 200444, People's Republic of China

## Abstract

Skin lesions not only disrupt appearance and barrier functionality but also lead to severe microbial infections and immune-inflammatory responses, seriously affect physical and mental health. *In situ* printing involves the direct deposition of bio-ink to create or repair damaged tissues or organs within a clinical setting. In this study, we designed and fabricated a novel portable *in situ* printer. This handheld instrument exhibits excellent printing performance, allowing hydrogels to be patterned and molded on surfaces according to specific requirements. By utilizing a dual-component hydrogels co-printing approach with high and low viscosities, we achieved *in situ* cell-laden printing using low-viscosity hydrogel. This demonstrates the advantages of the device in maintaining cell viability and achieving hydrogel structuring. This approach opens up the possibilities for the efficient encapsulation of active components such as drugs, proteins, and cells, enabling controlled macro- and micro-structuring of hydrogels. This breakthrough finding highlights the potential of our technical approach in dermatological treatment and wound repair, by dynamically adapting and regulating microenvironments in conjunction with hydrogel scaffolds and cell reparative impetus.

## I. INTRODUCTION

The skin serves as the primary barrier of the body, protecting internal organs and tissues from external damage, controlling body temperature, among other functions.^1–3^ However, it is also the organ most susceptible to external damage. Severe skin lesions such as rashes, acne, burns, scars, and wounds not only disrupt appearance and barrier functionality but also lead to severe microbial infections and immune-inflammatory responses.^4,5^ This significantly compromises both physical and mental health, imposing substantial economic burdens on both patients and society.^6–9^ Currently, common therapeutic approaches for skin disorders and injuries include oral pharmacotherapy (antibiotics, antifungals, and anti-inflammatories), topical medication (growth factors and corticosteroids), surgical interventions (lesion excisions, skin grafts, and laser therapy), and non-pharmacological treatments (dressings, wet compresses, and magnetotherapy).^8,10^ However, these methods all exhibit issues such as limited efficacy, medication side effects, adverse reactions, suboptimal therapeutic outcomes, and substantial inter-individual variability. Hence, in clinical practice, there is need for reliable treatment regimens that harness the body's innate self-repair mechanisms, thereby achieving the effective promotion of skin regeneration and wound healing.

Hydrogels have gained significant attention in the pharmaceutical and biomedical fields due to their various applications, such as drug delivery, cell therapy, and tissue engineering.^11,12^ With unique biocompatibility, tunable mechanical properties, and the ability to encapsulate viable cells, hydrogels exhibit promising potential in the field of skin repair.^13,14^ Within three-dimensional (3D) printing technology, hydrogels serve as a bio-ink, enabling precise layer-by-layer fabrication of hydrogel materials. This enables the incorporation of multiple bioactive components into distinct regions of the hydrogel construct, mimicking the natural cell microenvironment, promotes repair and regeneration in skin lesions.^15,16^ However, conventional bioprinting technology typically necessitates *in vitro* printing or culturing before implantation in the human body. When applying this technology to repair tissue or organ defects, accurately designing and manufacturing implants before surgery can be challenging due to uncertainties surrounding the shape of the defective tissue.^17,18^ *In situ* printing technology refers to a method that involves directly depositing biocompatible materials at tissue or organ defect sites to restore tissue integrity.^19^ Thus far, *in situ* printing technology has been used in applications such as skin, bone, and cartilage.^20,21^ Previous study demonstrated a handheld skin printer with a parallel multi-axis nozzle structure. This device facilitates the on-site creation of skin tissue sheets with varying structural components directly onto skin lesions, effectively replicating the structure of human skin, shown the feasibility and efficacy of *in situ* printing.^22^

Hydrogel materials alone cannot dynamically balance the inflammatory response of skin lesions and regulate the immune microenvironment, maintaining tissue balance.^23,24^ Delivering highly active cells has the potential to address the problem of low cell activity at defect sites and promote integration between scaffolds and hosts. Ideally, the viscosity of the hydrogels should support cell growth, differentiation, and bioprinting.^25^ However, in practice, a viscosity suitable for bioprinting might not support cell viability. The direct involvement of live cells restricts the broader utilization of biomaterials and the optimization of the printing process.^26–28^ Thus, to achieve optimal printability while ensuring high cell viability, it is necessary to optimize printing parameters and bio-ink consistency.

The application of portable *in situ* printing technology has the potential to enhance the clinical application of active components including medicines, proteins, and cells.^19^ Currently, two main methods of cell-laden hydrogel bioprinting are laser-assisted bioprinting and extrusion-based bioprinting.^29–31^ Laser-assisted bioprinting employs ultraviolet (UV) light and photo initiators, which can harm cells and limit clinical applicability due to utilize UV exposure.^31^ On the other hand, Cheng *et al.*^32^ employed an extrusion-based *in situ* printing method to directly print a fibroin hydrogel containing mesenchymal stem cells (MSC) onto full-thickness skin defects on pigs' dorsal region. This study successfully demonstrated epidermal and dermal regeneration, as well as vascular formation. While extrusion-based *in situ* bioprinting avoids UV exposure, it requires high-viscosity bio-inks for optimal printing outcomes. However, the use of high-viscosity hydrogel during printing can generate significant shear stress, potentially compromising cell viability and even causing cell death.^28,32,33^ Conversely, low-viscosity hydrogel might result in inadequate biomimetic scaffold structures and poor shape fidelity.^34,35^ The challenge of achieving both precise structural control and high cell viability simultaneously is a critical issue that needs to be addressed.

**Scheme 1.**
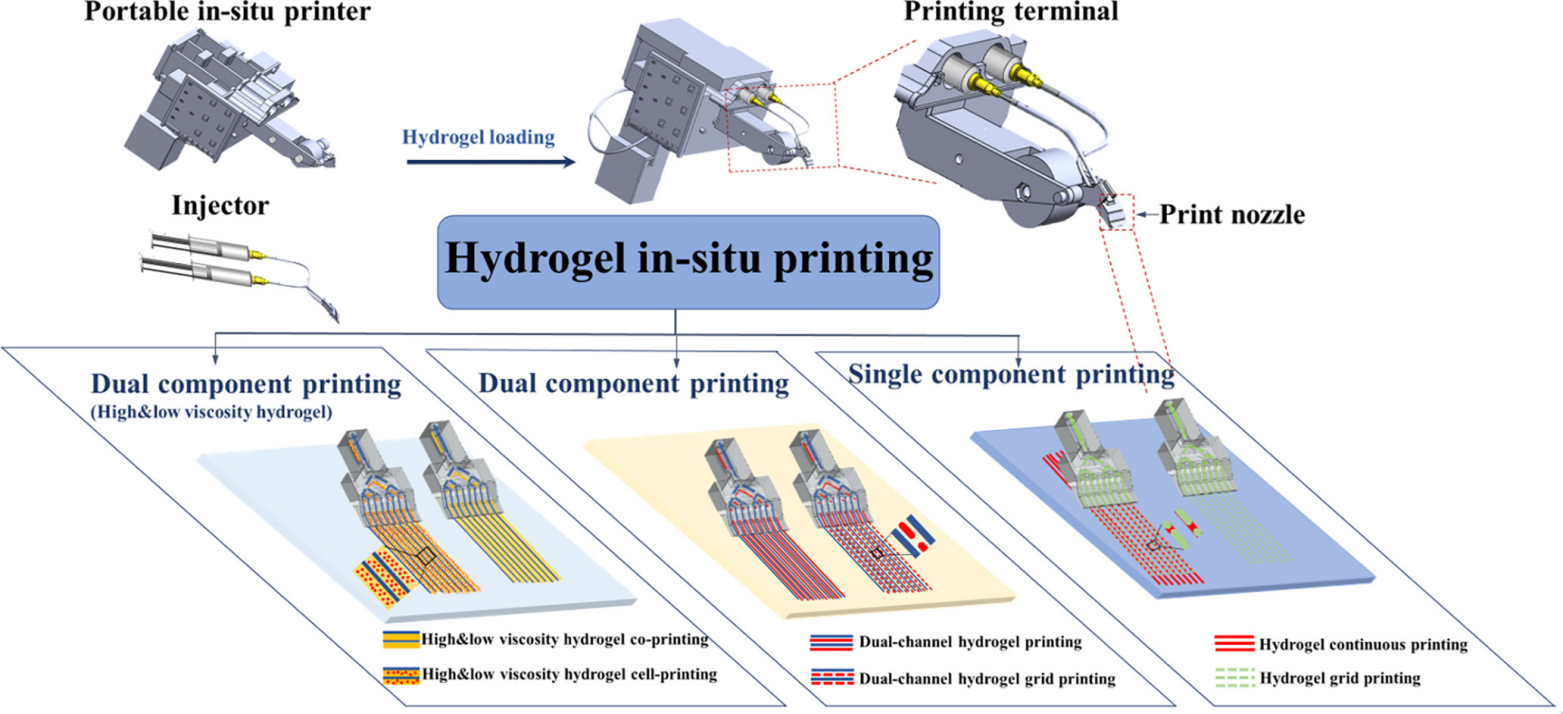
Schematic diagram for hydrogels printing preparation using a portable *in situ* printer. First, the hydrogel is loaded into a syringe, which is then placed into the handheld printer. The device is powered on, and the printing parameters are configured to match the extrusion speed driven by the stepper motor with the movement speed driven by the rollers. The print nozzle is positioned on the surface of the object to be printed, and the *in situ* printing is started. The printing process can be stopped by turning off the motor switch. The machine's parameters include various modes, and the stepper motor can achieve both continuous and intermittent propulsion. By collaborating with the nozzle, different hydrogel structures can be printed. (1) For single-component hydrogel, the printing methods include continuous printing with multi-channels, grid-like printing, and stacked printing. (2) Dual-component hydrogels can be printed using continuous printing with dual-component multi-channels, grid-like printing with dual-component multi-channels, and stacked printing with dual components. Importantly, this printing technique allows for the *in vitro* formation of low-viscosity hydrogel by using high-viscosity hydrogel dam to constrain the river formed by low-viscosity hydrogel. This enables cross-linking and molding of the hydrogels structure as required. This method is particularly suitable for cell-loaded printing using low-viscosity hydrogel.

Low-viscosity hydrogel generally ensures high cell viability, while high-viscosity hydrogels are more suitable for controlled macro- and microstructural molding.^36,37^ Striking a balance between these two factors is crucial for meeting the demands of skin lesion treatments using hydrogel *in situ* printing. *In situ* printing of hydrogels with different viscosities, where high-viscosity hydrogel helps shape low-viscosity ones, can enable the printing of cell-laden hydrogel with good viability and uniformity. Moreover, *in situ* printing allows for the application of active components in specific locations such as outdoor fields and emergency scenarios. Here, we have designed and assembled the device body, control circuitry, and control algorithms to create a portable *in situ* printer. This handheld printer allows for the patterned and controlled molding of hydrogels through parameter adjustments and a multi-channel print nozzle. To ensure successful cell printing and the survival of cell *in vivo*, we used a low-viscosity hydrogel as the medium for cell-loading. Additionally, we incorporated a high-viscosity hydrogel as a support layer, following the “dam–river” principle, to aid in the *in situ* molding of the low-viscosity hydrogel at the skin site. Our approach enables the bioprinting of hydrogels and cell-laden hydrogels suitable for *in vivo* application in large animals, and ultimately in clinical settings (Scheme 1).

## II. RESULTS AND DISCUSSION

### A. Assembling the portable *in situ* printer

**FIG. 1.**
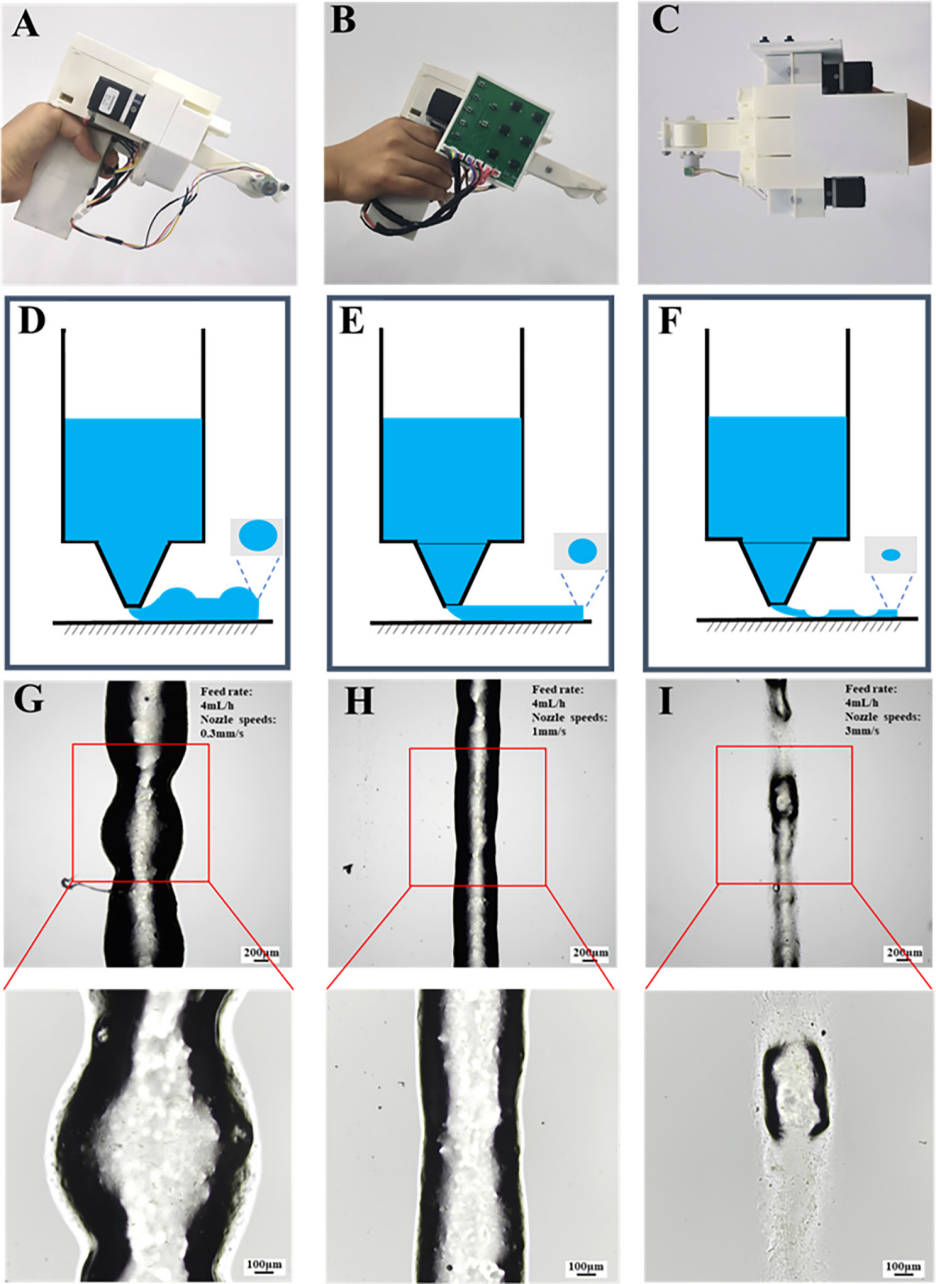
Portable *in situ* printer and hydrogels printing. Complete assembly diagram of the portable *in situ* printer in (a) front view, (b) rear view, and (c) top view. Schematic diagram of hydrogel *in situ* printing of (d) low-speed printing, (e) optimal-speed printing, and (f) high-speed printing. Hydrogel *in situ* printing in (g) low-speed printing, (h) optimal-speed printing, and (i) high-speed printing; the upper images show the original printing results (scale bar = 200 *μ*m), and the lower images provide localized magnification (scale bar = 100 *μ*m). The diagram in the upper right corner represents the hydrogel feed rate and nozzle movement speeds.

Conventional *in situ* printers consist of scanning, printing, and computer control systems. Printing systems that rely on a three-axis motion platform have a large footprint, complex operation, and indoor usage only, which restrict their adaptability to outdoor environments.^33,38,39^ In this study, we introduce a portable *in situ* printer, assembled from a main module and an extrusion and molding module, as shown in Figs. 1(a)–1(c). Two printed circuit board (PCB) boards are installed separately, one inside the handle and the other on the side of the device. This device is designed to be user-friendly and can be easily operated with just one hand.

Currently, handheld *in situ* printers based on electrospinning utilize high-voltage electrostatic forces to spin polymer solutions into nanofibers, but the presence of whipping effects prevents precise molding of these nanofibers.^40,41^ Furthermore, handheld *in situ* printer based on photopolymerization UV light to cross-link photosensitive hydrogels such as gelatin methacryloyl (GelMA) and hyaluronic acid methacrylate (HAMA) onto object surfaces.^42,43^ This printer use extrusion achieved hydrogels *in situ* molding, extrusion the hydrogels loaded into a syringe by mechanically forcing through a nozzle to form filaments.^44^ Notably, the printing process requires that the hydrogels extrusion speed matches the nozzle printing speed. Figures 1(d)–1(f) represent schematic diagrams of different printing results. If the printing speed is lower than the optimal printing speed, there is an accumulation of hydrogel materials, resulting in hydrogel fibers with diameters larger than the nozzle diameter [Fig. 1(d)]. Conversely, when the printing speeds exceed the optimum printing speed, the hydrogel fibers undergo stretching, causing a reduction in fiber diameter and potential for fracture [Fig. 1(f)]. Only when the extrusion speed is aligned with the optimal printing speed, do hydrogels fiber diameter closely match the nozzle diameter, yielding the highest print quality [Fig. 1(e)].

The extrusion module of the printer utilizes a roller to enhance printing stability by propelling the movement of the nozzle, through regulating the nozzle's movement speed to achieve hydrogels printing. To evaluate the print stability and operability of the printer, we observed pre-crosslinked 4% Alg hydrogel printing under different speed conditions. The hydrogel's feed rate was set at 4 ml/h, and the nozzle's movement speeds were set at 0.3, 1, and 3 mm/s. With a nozzle's movement speed of 0.3 mm/s, hydrogel fibers exhibit accumulation and assume an elliptical shape [Fig. 1(g)]. When the nozzle's movement speed is 1 mm/s, the fiber diameter closely matches the nozzle diameter, resulting in a cylindrical shape [Fig. 1(h)]. However, when the nozzle's movement speed of 3 mm/s, the extruded fiber diameter becomes finer and tends to exhibit fractures [Fig. 1(i)]. In conclusion, it is crucial to achieve a balance between the feed rate and printing speeds for successful extrusion printing.

### B. Dual-component multi-nozzle

While this printer excels in achieving stable hydrogels printing, the printing efficiency of the single-channel nozzle remains relatively low, which fails to meet practical demands.^42^ Furthermore, it lacks the capability to achieve precise shaping of different component hydrogels. To address these limitations and enhance the efficiency of hydrogels printing, we have developed independent multi-channel hydrogel printing nozzles [supplementary material Fig. S1(a)]. Two identical initial channels with multiple latter channels are staggered, allowing for independent control of hydrogel molding.

During the molding experiments conducted with the aforementioned dual-component multi-nozzle, different material's feeding mode settings yield three distinct printing outcomes, as illustrated in supplementary material Figs. S1(b) and S1(c). Utilizing a single channel enables the formation of an array-patterned hydrogel fiber, significantly enhancing printing efficiency [supplementary material Fig. S1(b)]. By utilizing both channels for printing, it allows for alternating deposition of different-component hydrogel materials [supplementary material Fig. S1(c)]. This molding effect effectively addresses the demands of diverse hydrogel composite printing, and the method supports the maximum achievable molding of various hydrogel types within this alternating distribution structure. Furthermore, simultaneous printing through both channels, with one channel intermittently feeding, yields a single-layer grid-like hydrogels structure [supplementary material Fig. S1(d)].

Simulation and modeling of hydrogels extrusion printing within the nozzle were carried out using COMSOL software to investigate the extrusion behavior of hydrogels with varying viscosities. The simulation outcomes for hydrogels with viscosities of 220 and 1000 mPa s, at initial velocities of 0.2, 15, and 30 mm/s, are illustrated in supplementary material Figs. S1(e) and S1(f), respectively. Upon analyzing the simulation outcomes, it is evident from the velocity distribution maps on the left side that the hydrogel attains its highest velocity at the inlet and gradually decelerates. The linear velocity distribution profiles on the right-hand side reveal a relatively uniform velocity distribution at each exit point, with minimal discrepancies. These prominent features are consistent across simulations involving hydrogels of both high and low viscosities. These experimental findings support the nozzle's ability to effectively print composite hydrogels with varying viscosities.

### C. *In situ* printing with dual-component multi-nozzle

**FIG. 2.**
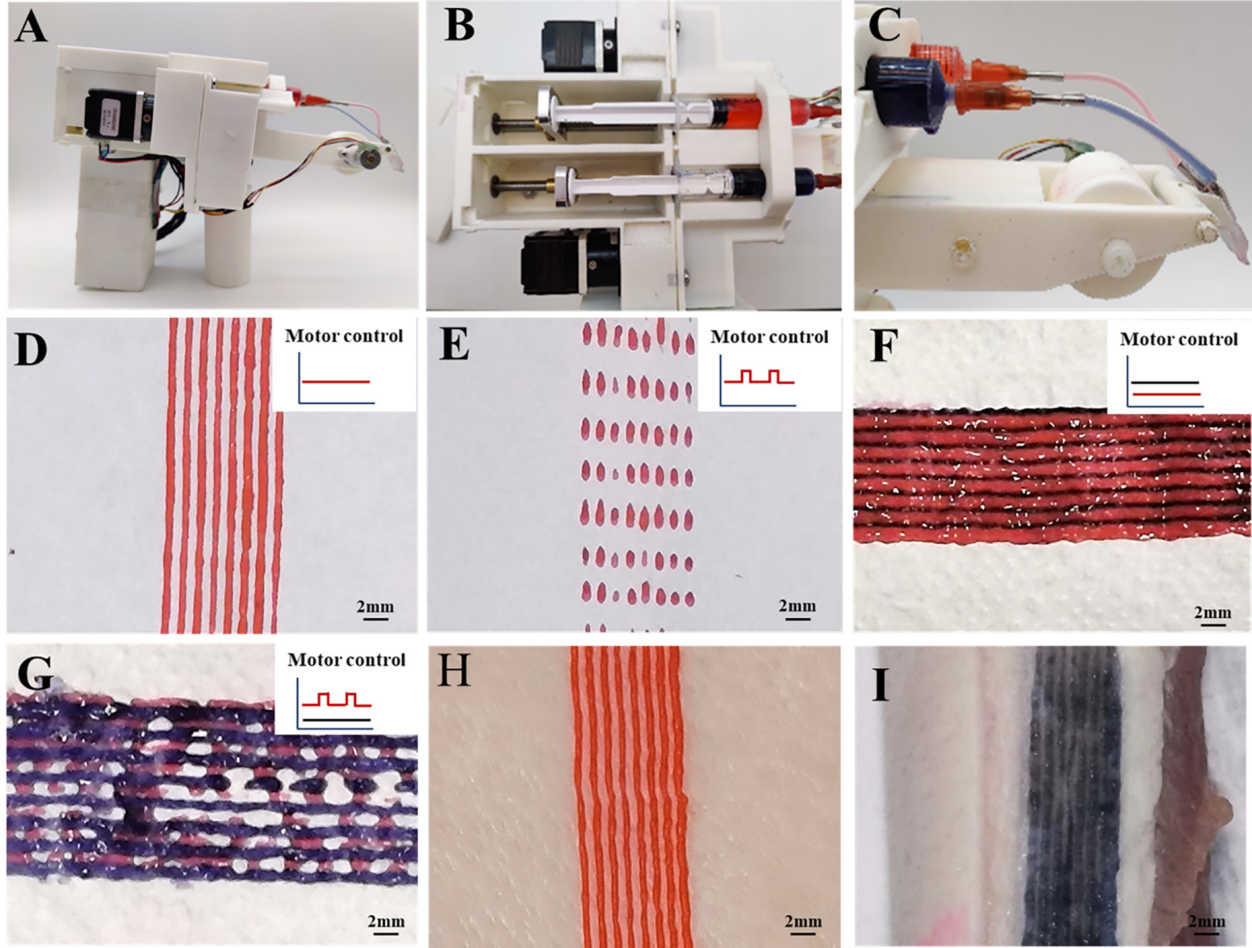
*In situ* printing of various hydrogel structures using the portable *in situ* printer. Assembly of the nozzle and hydrogel for the portable *in situ* printer in (a) whole, (b) main body module, and (c) extrusion and molding module. *In situ* printing of various hydrogel structures of (d) continuous printing of single-component multi-channels, (e) grid-like printing of single-component multi-channels, (f) continuous printing of dual-component multi-channels, and (g) grid-like printing of dual-component multi-channels. The diagram in the upper right corner illustrates the waveform of motor control. (h) Complete continuous printing of single-component multi-channels on pig skin; and (i) complete continuous printing of single-component multi-channels on pig meat (scale bar = 2 mm).

We equipped a composite print head onto a portable *in situ* printer and evaluated the printing outcomes. Hydrogels printing parameters were skillfully managed by controlling the feeding mode of the stepper motor [Figs. 2(a) and 2(b)]. The attachment of the print head to the extrusion module was achieved through the use of a fixture [Fig. 2(c)]. We tested the printer's on-site printing ability for different hydrogel structures. By adjusting the stepper motor to continuous mode, we were able to continuously print single-component hydrogel using multiple channels [Fig. 2(d)]. Switching the stepper motor to intermittent mode enabled grid-like printing of single-component hydrogel through multiple channels [Fig. 2(e)]. Employing continuous mode for both stepper motors facilitated continuous printing of dual-component hydrogels [Fig. 2(f)], while configuring one stepper motor in continuous mode and the other in intermittent mode allowed grid-like printing of dual-component hydrogels [Fig. 2(g)]. It is crucial to match the printing process parameters with the materials in order to achieve desired patterned shaping.

Skin has emerged as an ideal testing ground for *in situ* bioprinting investigations.^45^ *In situ* bioprinting with hydrogels could prove effective in skin disease and wound healing.^38,46^ We validated the printing performance of this printer on pig skin and pork, demonstrating its capability for continuous hydrogels printing on these surfaces [Figs. 2(h) and 2(i)]. However, hydrogel fibers exhibited discontinuity when printed *in situ* on pork, potentially due to the unevenness of the pork surface, preventing stable nozzle-pork alignment. In summary, this printer has the capability to perform printing on both the surface and internal layers of the skin, thanks to its multi-nozzle setup that enables accurate and efficient drug deposition on the skin. This technology has immense potential for rapidly closing lesions and holds great promise in the field of skin disease and wound treatment.^22^ In the future, there will be a need for more agile and user-friendly equipment to address the treatment requirements of specific anatomical regions with defects and pathologies.

### D. *In situ* printing with different viscosity hydrogels

**FIG. 3.**
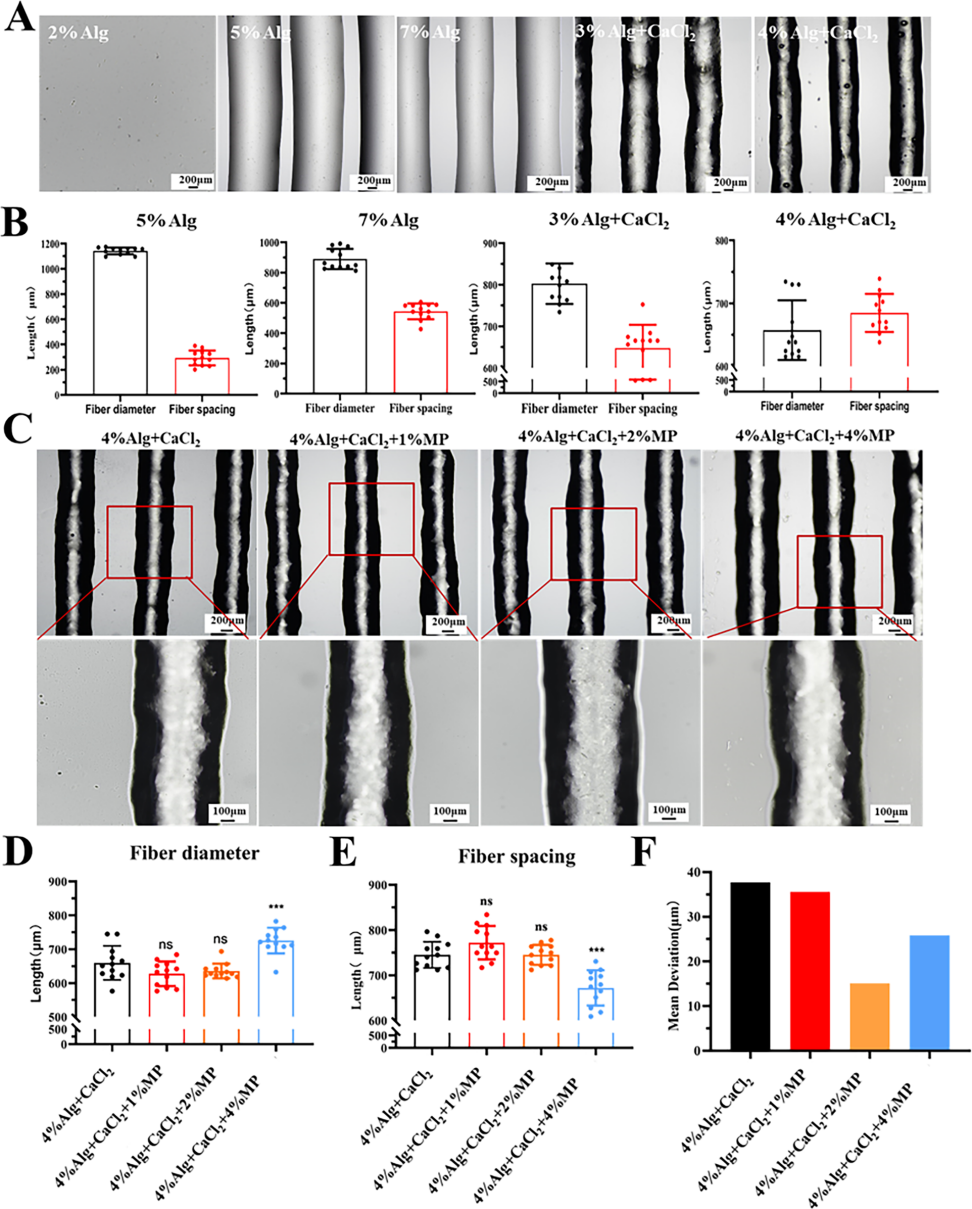
Hydrogel *in situ* printing outcomes. (a) Molding of sodium alginate (Alg) hydrogel with different concentrations and cross-linking, including 2% Alg, 5% Alg, 7% Alg, 3% Alg with calcium chloride (CaCl_2_) pre-cross-linking, and 4% Alg with CaCl_2_ pre-cross-linking. (b) The fiber diameter and spacing were measured after molding of Alg hydrogel with different concentrations and cross-linking, including 2% Alg, 5% Alg, 7% Alg, 3% Alg + CaCl_2_, and 4% Alg + CaCl_2_. (c) 4% Alg hydrogels were molded with different concentrations of mussel protein (MP) enhancement, including 0% MP, 1% MP, 2% MP, and 4% MP. The upper images show the printing results (scale bar = 200 *μ*m), and the lower images provide localized magnification (scale bar = 100 *μ*m). (d) The fiber diameter in Alg hydrogel was measured with different concentrations of MP. (e) The spacing between fibers in Alg hydrogel was measured with different concentrations of MP. (f) The average difference in fiber diameter of Alg hydrogel was calculated with different concentrations of MP. Data are presented as means ± standard deviation (SD). ^***^p < 0.001.

When assessing the printability of biomaterials, shape fidelity and printing resolution are crucial considerations.^47,48^ Alginate (Alg) is a widely employed bio-ink material due to its cellular compatibility, cost-effectiveness, availability, and printability.^49,50^ To investigate the printing capabilities of the printer using hydrogels with different viscosities, we employed Alg hydrogel with varying concentrations and pre-crosslinked levels. Printing was executed through a nozzle with a 600 *μ*m inner diameter, and the extrusion printing results are illustrated in Fig. 3(a). The 2% Alg hydrogel exhibited inability to form upon extrusion, and both the 5% and 7% Alg hydrogel effectively maintained their fibrous structure immediately after printing. Pre-crosslinked 3% Alg hydrogel and 4% Alg hydrogel sustained their fibrous architecture even after an extended post-printing period. The diameter distribution of the different hydrogel fibers is depicted in Fig. 3(b). Specifically, the fiber diameter of 5% Alg hydrogel was measured to be 1142.99 ± 27.65 *μ*m, 7% Alg hydrogel was 890.06 ± 67.48 *μ*m, pre-crosslinked 3% Alg hydrogel was 802.91 ± 48.6 *μ*m, and the pre-crosslinked 4% Alg hydrogel was 657.40 ± 47.35 *μ*m. The fibrous morphology of the 4% Alg hydrogel closely resembled the inner diameter of the nozzle, indicating favorable printing performance. However, due to the collapsible and expansible characteristics of hydrogel fibers,^51^ observing the printing results, structural collapse of hydrogels post-extrusion led to reduced straightness of their boundaries, resulting in weaker formation.

To further investigate the impact of different hydrogel strengths on printing outcomes and validate the printer's inherent performance, we assessed the printing effects of hydrogels with varying strengths. MP (mussel adhesive protein) could forms polymers through self-oxidation cross-linking of dopamine groups, with increase in molecular weight. It provides important benefits for promoting cell proliferation, enhancing cell adhesion, and accelerating wound healing.^52–54^ Through the incorporation of MP into Alg, we effectively augmented the strength of the resulting hydrogel, consequently observing enhancements in its printing performance. Our experiments confirmed that the Alg hydrogel, enriched with MP, could be successfully extruded and molded [Fig. 3(c)]. Stiffness measurements were conducted using an atomic force microscope (AFM), revealing Young's modulus values for hydrogels of 196.33 ± 27.38, 503.00 ± 43.46, 651.67 ± 15.33, and 930.33 ± 74.21 kPa, respectively (Fig. S2). These results indicate that the mechanical stiffness of the hydrogels increased as the MP concentration increased. Furthermore, we performed an analysis of the extruded fiber diameter and spacing. For pre-crosslinked 4% hydrogel at concentrations with 0% MP, 1% MP, 2% MP, and 4% MP, the measured fiber diameters were 659.45 ± 50.03, 627.43 ± 36.15, 635.63 ± 21.55, and 725.43 ± 37.79 *μ*m, respectively. Notably, hydrogel fibers displayed larger diameters at a 4% MP concentration. There were no statistically significant differences in extruded fiber diameters among hydrogels with concentrations of 0% MP, 1% MP, and 2% MP. The inter-fiber spacing within the hydrogel fibers was also evaluated. Hydrogel fibers at a 4% MP concentration exhibited finer spacing of 672.33 ± 39.07 *μ*m [Figs. 3(c) and 3(d)]. Furthermore, a comparison of the mean differences in fiber diameter among different hydrogel compositions revealed that 2% MP displayed a smaller average difference of just 15.10. These findings highlight the improved printing performance achieved with 2% MP and emphasize the significance of using suitably reinforced hydrogels to enhance printing efficiency.

To enhance the therapeutic effectiveness of hydrogels, incorporation bioactive components such as growth factors, signaling proteins, and cells within biomaterials are a viable strategy. With the rise of *in situ* printing, there is an increasing potential to utilize stimulus-responsive smart materials for the fabrication of biomimetic structures. These smart materials can be controlled dynamically through external interventions such as electric currents, magnetic fields, and light, allowing for the on-site regeneration of tissues.

### E. *In situ* bioprinting of cell-laden hydrogel

Cells play a crucial role in the regeneration of tissues. Keratinocytes, fibroblasts, and MSC are commonly used in wound healing and dermatological treatments.^55^ By incorporating cells within hydrogels, a higher concentration of active cells can be localized to the treatment area, which can accelerate wound healing and dermatological recovery, facilitating the growth and regeneration of new tissue. Extrusion printing enables the achievement of higher cell densities, especially when dealing with materials of high viscosity (above 6 × 10^7^ mPa/s).^56^ Moreover, combining different types of cells within the hydrogels can enhance intercellular communication and interactions, leading to an overall improvement in wound repair and dermatological treatment outcomes.

**FIG. 4.**
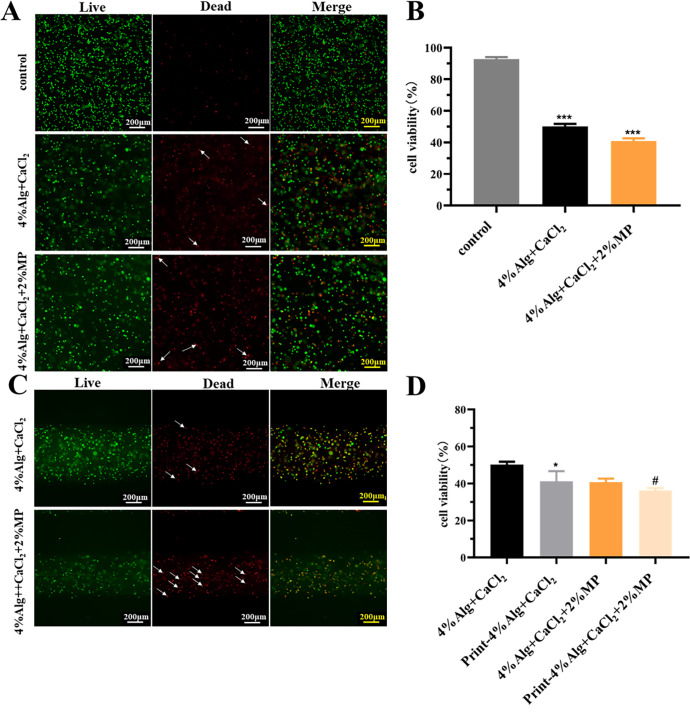
Cell-loaded Alg hydrogel *in situ* printing. (a) Viability of 293T cells before hydrogel printing assessed using live/dead staining (scale bar = 100 *μ*m). (b) Statistics of 293T cell viability before hydrogel printing. (c) Viability of 293T cells after hydrogel printing assessed using live/dead staining (scale bar = 200 *μ*m). (d) Statistics of 293T cell viability after hydrogel printing. White arrows in the images indicate distorted dead cells. Data are presented as means ± SD. ^*^ and ^#^p < 0.05, ^***^p < 0.001.

We mixed 293T cells into the hydrogels matrix and analyzed cell viability within Alg hydrogel to investigate the efficacy of *in situ* bioprinting employing extrusion-based techniques with cell-laden hydrogels. The results showed that the viability prior to printing in pre-crosslinked 4% Alg (50.20 ± 1.60%) and pre-crosslinked 4% Alg with 2% MP (40.84 ± 1.81%) was significantly lower than the control group (92.87 ± 1.13%). Furthermore, several cells displayed distortion and cell death [Figs. 4(a) and 4(b)]. This phenomenon might be attributed to the mechanical stress and pressure exerted on cells by high-viscosity hydrogels, leading to cellular compression and consequent mortality, thereby reducing cellular activity.

Cell-laden hydrogels could extrude through the nozzle to produce fibers with homogeneous cell distribution and favorable fiber morphology. However, a significant number of cells within the hydrogel were found to be nonviable, with the majority undergoing cell death and deformation, particularly in the pre-crosslinked 4% Alg with 2% MP [Fig. 4(c)]. Furthermore, analysis showed that post-printing cell viability for the pre-crosslinked 4% Alg (41.26 ± 4.43%) was lower than the preprinting cell viability (50.20 ± 1.30%), and for the pre-crosslinked 4% Alg with 2% MP, post-printing cell viability (36.36 ± 1.02) was lower than the preprinting (40.84 ± 1.48%) [Fig. 4(d)]. The previous study shows that the extrusion printing procedure had a negative influence on cell viability due to cell damage caused by shear stress throughout the process. This effect was affected by variables such as printing pressure, nozzle diameter, and length.^57,58^ Particularly, noteworthy is that high-viscosity hydrogel often required high pressure to be successfully printed through micro-size nozzles. However, increased pressure can potentially cause further harm to cells, exacerbating the damage.

### F. *In situ* bioprinting of dual-component hydrogels with varying viscosities

An ideal hydrogel viscosity should not only support cell growth and differentiation but also be suitable for printing.^25,47^ However, in reality, hydrogel viscosities suitable for bioprinting may not adequately support cell functions (proliferation, migration, differentiation, and secretion).^28,59^ Our findings indicate that high-viscosity hydrogel suitable with printing might cause significant cell damage, suggesting the potential need for low-viscosity hydrogel in cell-laden printing processes. Nevertheless, the currently employed photopolymerization printing methods struggle with low-viscosity materials' precise structuring, controlled light intensity, and exposure duration, which impede the regulation of printed structure mechanical properties and the creation of intricate architectures.^43,60^ To ensure structural fidelity, secondary cross-linking through external environmental signals such as UV radiation curing, chemical exposure, and temperature change is necessary.^61–63^ This process takes a long time are incompatible with *in situ* bioprinting because they cannot be introduced consistently into the living organism while surgical operations are being performed. Therefore, we need to explore the use of extrusion-based printing technologies to enable the creation of low-viscosity hydrogel on printed surfaces on-site.

**FIG. 5.**
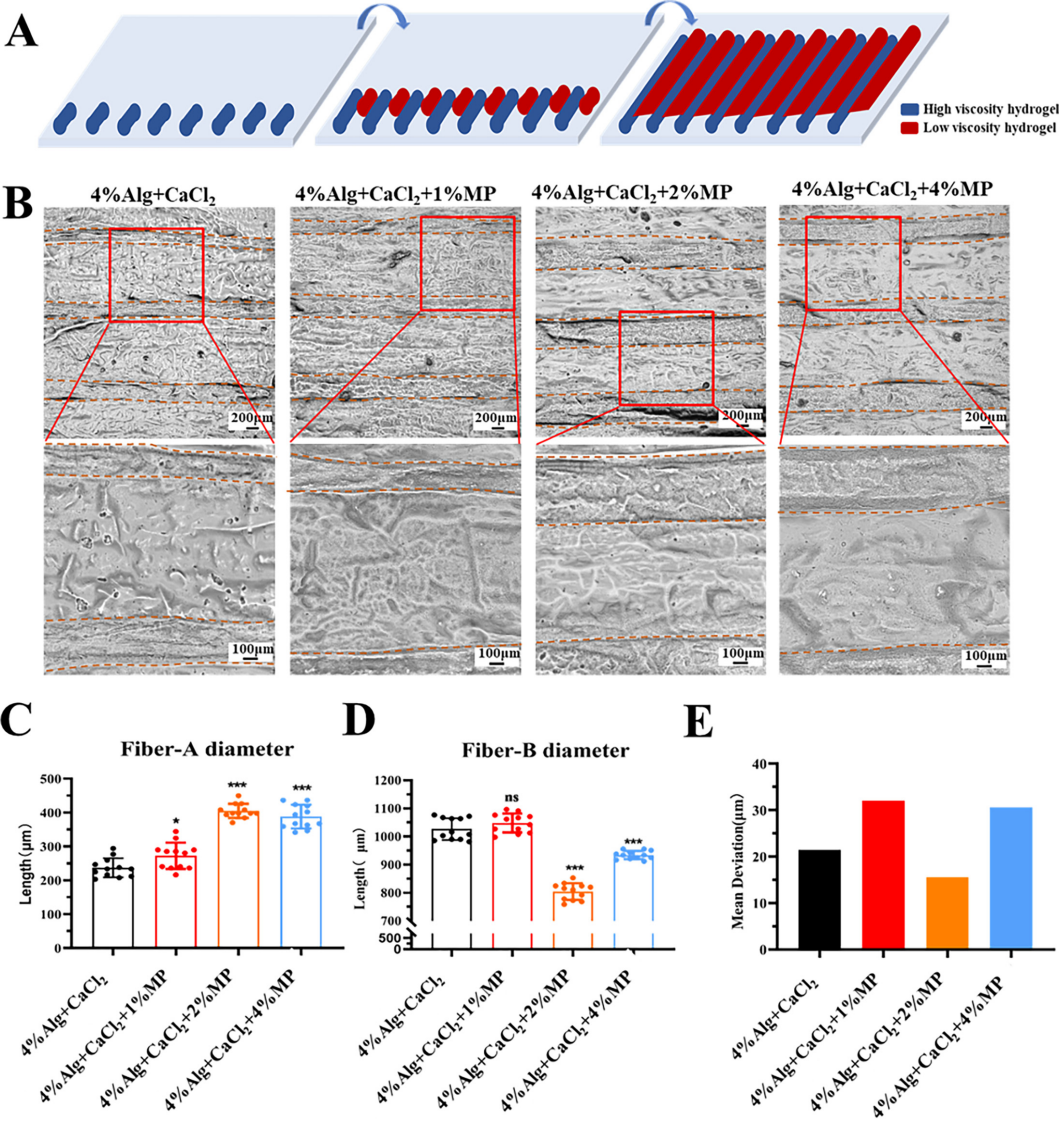
*In situ* printing of high- and low-viscosity dual-component hydrogels. (a) Schematic diagram of *in situ* printing with high- and low-viscosity dual-component hydrogels: blue represents high-viscosity hydrogel (dam); red represents low-viscosity hydrogel (river). (b) The molding of dual-component hydrogels using a combination of high-viscosity hydrogel (dam) and low-viscosity hydrogel (river). Various concentrations of Alg + CaCl_2_ were used for the high-viscosity hydrogel (dam) (4% Alg + CaCl_2_, 4% Alg + CaCl_2_ + 1% MP, 4% Alg + CaCl_2_ + 2% MP, 4% Alg + CaCl_2_ + 4%MP), while the low-viscosity hydrogel (river) was kept at a constant concentration of 2% Alg. The upper images show the outcomes of the printing process (scale bar = 200 *μ*m), while the lower images provide a magnified view (scale bar = 100 *μ*m). The shorter distances between the brown dashed lines represent the fibers of the high-viscosity hydrogel (dam), while the longer distances indicate the fibers of the low-viscosity hydrogel. (c) Fiber diameter of high-viscosity hydrogel (dam) with different concentrations of MP. (d) Fiber diameter of low-viscosity hydrogel (river). (e) Average difference in fiber diameter of high-viscosity hydrogel (dam). Data are presented as means ± SD. ^*^p < 0.05, ^***^p < 0.001.

To overcome the challenges associated with *in situ* molding of low-viscosity hydrogel, we utilized a dual-component nozzle for composite printing. This allowed us to print high- and low-viscosity hydrogel together, enabling on-site molding of the low-viscosity hydrogel. In this printing process, the high-viscosity hydrogel acted as a “dam” to hold the low-viscosity hydrogel (river) within the gaps of the high-viscosity hydrogel fibers. Resulting in the *in situ* molding of the low-viscosity hydrogel structure [Fig. 5(a)], we conducted experiments using four different types of high-viscosity hydrogel: pre-crosslinked 4% Alg, pre-crosslinked 4% Alg with 1% MP, pre-crosslinked 4% Alg with 2% MP, and pre-crosslinked 4% Alg with 4% MP. The low-viscosity hydrogel (river) remained constant at 2% Alg. Despite the challenge posed by the high fluidity of the low-viscosity hydrogel, causing it to spread, our results showed successful molded of both high- and low-viscosity hydrogel [Fig. 5(b)].

Furthermore, analysis was conducted on the diameters of high- and low-viscosity hydrogel fibers. The diameters of high-viscosity hydrogel fibers were measured, pre-crosslinked 4% Alg with 2% MP (404.8 ± 21.09 *μ*m) and pre-crosslinked 4% Alg with 4% MP (388.01 ± 34.98 *μ*m), were found to be higher than the control group with pre-crosslinked 4% Alg (237.06 ± 28.07 *μ*m) [Fig. 5(c)]. Among these, the larger diameters of the low-viscosity hydrogel fibers, specifically pre-crosslinked 4% Alg with 2% MP (804.30 ± 29.59 *μ*m) and pre-crosslinked 4% Alg with 4% MP (934.18 ± 14.80 *μ*m), had lower diameters compared to the control group with pre-crosslinked 4% Alg (1026.4 ± 138.64 *μ*m) [Fig. 5(d)]. The fiber diameter of low-viscosity hydrogel is much larger than that of high-viscosity hydrogel. This phenomenon occurs because the high-viscosity hydrogel forms a dam that is then fixed with the low-viscosity hydrogel. As a result, the low-viscosity hydrogel forms a “river” that “submerges” the dam. Notably, the addition of 2% ovalbumin to the high-viscosity hydrogel resulted in a diameter of 404.8 ± 21.09 *μ*m, significantly larger than the 237.06 ± 28.07 *μ*m observed for pre-crosslinked 4% Alg, with an average difference of 15.55 [Fig. 5(e)]. The results indicated that the pre-crosslinked 4% Alg with 2% MP exhibited favorable shaping effects. The presence of MP loaded within the high-viscosity hydrogel might have indirectly influenced the strength and shaping of the hydrogel. A recent study has showcased *in situ* printing on the skin using various materials. For instance, GelMA was successfully utilized with a molding time of 20 s,^43^ GelMA/HAMA with molding time of 10 s,^60^ and Alg and fibrinogen composites with molding times ranging from 44 to 160 s.^22^ Similarly, a combination of hyaluronic acid (HA) and fibrinogen achieved formation in 4.2 min.^32^ Although light cross-linking results in shorter formation times, low-viscosity hydrogel experienced structural changes within a brief period, preventing the sustained maintenance of the initial printed shape. This emphasizes the challenge of maintaining immediate print morphology with low-viscosity hydrogel. Our study shows that *in situ* printer is capable of rapidly *in situ* printing high-strength materials like Alg hydrogel and without the need for secondary cross-linking. Low-viscosity hydrogel can be surrounded by high-viscosity hydrogel on-site and subsequently undergo *in situ* cross-linking and molding using chemical agents, light, heat, enzymes, and other methods.

### G. *In situ* cell bioprinting dual-component high- and low-viscosity hydrogel

To explore the benefits of using dual-component high- and low-viscosity hydrogel *in situ* cell bioprinting for printing cell-laden structures, we load 293 cells into the low-viscosity Alg hydrogel to improve cell viability throughout the printing procedure.

**FIG. 6.**
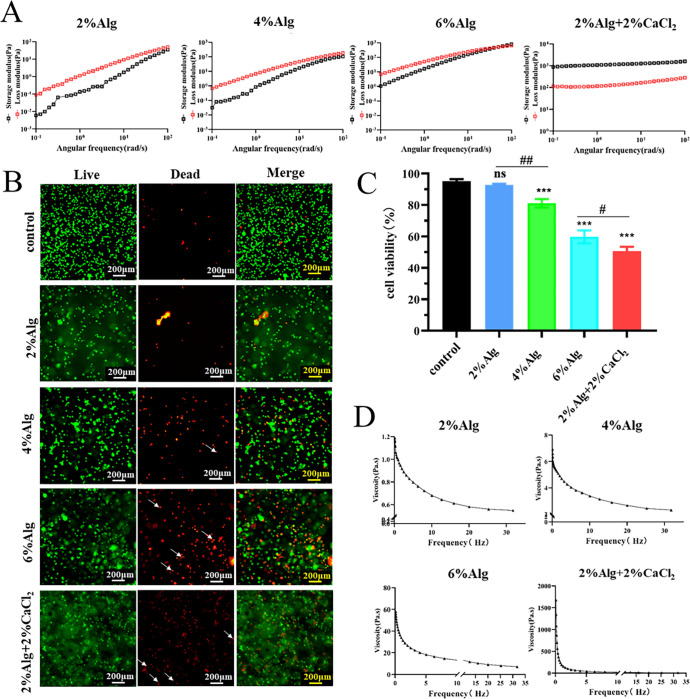
Cell viability in different viscosity hydrogels. (a) Rheological analysis of hydrogels. Frequency sweep analysis ranging from 0.1 to 100 rad/s. The storage modulus (G′) is represented by black squares, while the loss modulus (G″) is represented by red squares. (b) Viability of 293T cells in different hydrogels assessed using live/dead staining. Scale bar = 200 *μ*m. (c) Statistics of 293T cells viability. (d) Viscosity of different hydrogels. White arrows in the images indicate distorted dead cells. Data are presented as means ± SD. ^*^ and ^#^p < 0.05, ^##^p < 0.01, ^***^p < 0.001.

Cell viability in different hydrogel concentrations was analyzed prior to cell-laden printing. Initially, low-concentration Alg hydrogel was prepared. Throughout the frequency sweep, the storage modulus (G′) of 2%, 4%, and 6% Alg was lower than the loss modulus (G″), indicating that weakly crosslinked low-concentration Alg hydrogel lacked the strength to maintain fiber integrity after printing. Pre-crosslinked 2% Alg hydrogel consistently exhibited higher G′ than G″, indicating a robust crosslinked structure that could integrity fiber integrity post-printing [Fig. 6(a)]. Subsequently, live/dead staining results demonstrated robust cell viability within 2% Alg and 4% Alg hydrogel, higher than 80%. Notably, cell distribution within the low-viscosity hydrogel was notably even, particularly evident in the 2% Alg hydrogel, where cell viability (95.18 ± 1.25) showed no significant deviation from the control group (95.18 ± 1.25) [Figs. 6(a) and 6(c)]. Furthermore, the viscosity of the hydrogels was assessed at 10 rad/s, yielding viscosities of Alg hydrogel was 0.68, 3.39, 13.11, and 23.93 Pa s [Fig. 6(d)], respectively. All samples exhibited shear-thinning behavior, indicating non-Newtonian pseudoplastic fluid characteristics.^64^ Shear thinning behavior in extrusion printing is advantageous as it reduces dispensing pressure during printing, which is beneficial for maintaining cell viability.^65^ This means that when the hydrogel viscosity exceeds 3.39 Pa s (10 rad/s), cell viability significantly decreases below 85%. Conversely, when the viscosity is below 0.68 Pa s (10 rad/s), cell viability is notably higher, exceeding 90%. The survival rate of cells within low-viscosity hydrogel is significantly greater than that within high-viscosity hydrogel [Figs. 6(c) and 6(d)].

**FIG. 7.**
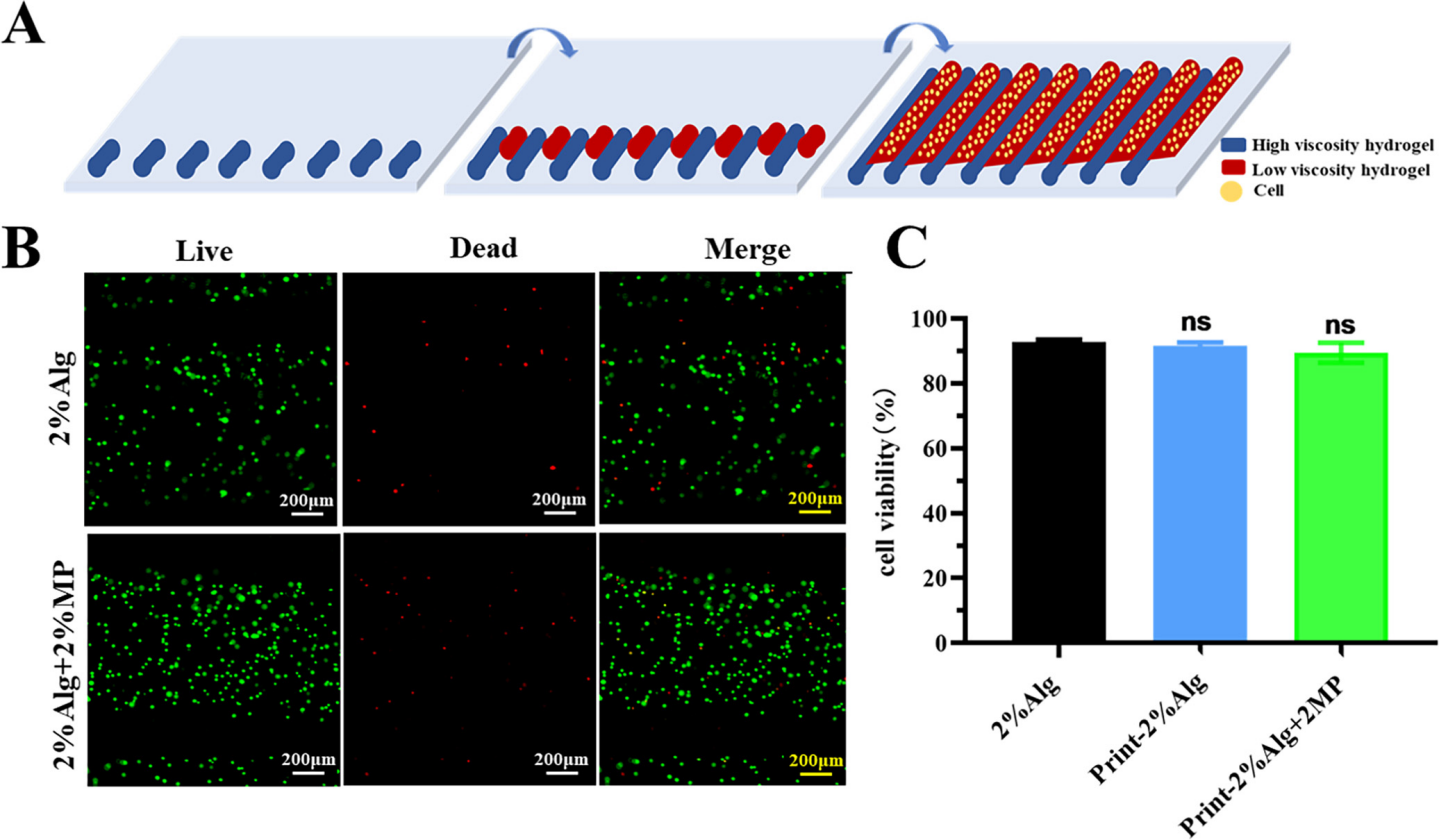
*In situ* cell printing of high- and low-viscosity dual-component hydrogels. (a) Schematic diagram of *in situ* cell printing with high- and low-viscosity dual-component hydrogels. Blue represents high-viscosity hydrogel (dam); red represents low-viscosity hydrogel (river); yellow represents cells. (b) Viability of 293T cells after hydrogels printing assessed using live/dead staining. Cell-loaded low-viscosity hydrogel (river) with 2% Alg and 2% Alg + 2%MP (scale bar = 200 *μ*m). (c) Statistics of 293T cells viability after hydrogel printing. Data are presented as means ± SD.

Cells were mixed into a composite hydrogel consisting of high- and low-viscosity hydrogel using Alg hydrogel for printing. The high-viscosity hydrogel acted as a dam to anchor the low-viscosity hydrogel (pre-crosslinked Alg), creating a river-like structure [Fig. 7(a)]. The cells were evenly distributed in both 2% Alg and 2% Alg with 2% MP. After printing, the cells were found within the low-viscosity hydrogel, separated by the high-viscosity fibers, forming strands of cell-laden hydrogel fibers. Furthermore, analysis showed that cell viability rates were higher in both the 2% Alg (91.62 ± 1.00%) and the 2% Alg with 2% MP (89.48 ± 3.09%), with no significant difference between them. Additionally, there was no significant change in cell activity before and after printing [Figs. 7(b) and 7(c)]. Cell viability (>90%) was higher than traditional extrusion printing (40%–80%).^33^

This bioprinting approach preserves cell viability without any physical or chemical changes, and it can print cells without compromising cell viability. In extrusion printing, application of optoelectronic technologies such as piezoelectric and laser methods is employed to achieve high-resolution microdroplets and microfibers, allowing for the creation of intricate and finely detailed structures.^66–68^ Techniques such as photocuring and laser assistance enable contactless printing without the need for nozzles, which minimizes any potential damage to the cells.^45,69^ Furthermore, the incorporation of technologies like infrared fluorescence enables real-time adjustments during printing, allowing for the customization of tissues with higher precision based on clinical requirements.^56,70,71^ This technology facilitates the precise replication of multi-layered cell-load skin, holding significant promise for the advancement of skin printing in the future.

## III. CONCLUSION

In summary, we have designed and fabricated a novel portable *in situ* printer. This handheld instrument exhibits excellent printing performance, allowing hydrogels to be patterned and molded on surfaces according to specific requirements. Through the dual-component high- and low-viscosity hydrogel co-printing approach, we achieved *in situ* cell-laden printing using low-viscosity hydrogel. This demonstrates the device's advantages in maintaining cell viability and achieving hydrogel structuring. It opens up the possibilities to efficient encapsulation of active components such as drugs, proteins, and cells, enabling controlled macro- and micro-structuring of hydrogels. This breakthrough finding highlights the potential of our technical approach in dermatological treatment and wound repair, by dynamically adapting and regulating microenvironments in conjunction with hydrogel scaffolds and cell reparative impetus.

## IV. MATERIALS AND METHODS

### A. Portable *in situ* printer

#### 1. Device design

The structure of the portable *in situ* printer was designed using SolidWorks 2020 software (Dassault Systèmes, Massachusetts, USA). The mechanical configuration consists of two detachable parts, as illustrated in supplementary material Fig. S3, the main body module [supplementary material Figs. S3(a) and S3(b)] and the extrusion module [supplementary material Fig. S3(c)]. The main body module comprises components for material feed propulsion, power transmission for motion, space for the core logic control board, a grip structure, and a keypad for adjusting printing parameters.

Upon assembling the extrusion and molding module with the main body module, the *in situ* extrusion process for the hydrogels can be performed. The handheld *in situ* printer [supplementary material Figs. S3(d)–S3(f)], once assembled, allows effective control of the nozzle movement speed through the motor-driven roller.

#### 2. Control system

Two stepper motors integrated within the device are employed to manage material feed speed and mode. One stepper motor controls the movement speed of the nozzle, while the actions of three motors are governed by the control system (STM32C8T6 core board). An overview of the control system architecture is depicted in supplementary material Fig. S4(a).

The core logic mainboard printed circuit board (PCB) facilitates connections between the STM32C8T6 core control board and the stepper motor driver board. The circuit schematic of this board is illustrated in supplementary material Fig. S4(b). The keypad interface PCB integrates the keypad buttons for sending STM32C8T6 control signals and motor switches. The circuit schematic for this keypad is presented in supplementary material Fig. S4(c). The entire control system principle diagram is presented in supplementary material Fig. S5.

#### 3. Integral assembly

The structural support components of the device are fabricated using a Raise3D E2 desktop fused deposition modeling (FDM) 3D printer (Raise3D, Shanghai, China), utilizing polylactic acid (PLA) as the printing material. Once all the standardized components, encompassing the PCB boards and structural components, the entire assembly process follows the design model to create the portable *in situ* printer.

### B. Printing nozzle

#### 1. Nozzle design

The structure of the nozzle was designed using SolidWorks 2020 software (Dassault Systèmes, Massachusetts, USA). The nozzle features two separate hydrogel printing channels. Hydrogels entering through a single inlet and passing through a complex network of channels before exiting from eight outlets spaced at specific intervals. By arranging two identical sets of these printing channels in a staggered configuration and offsetting them vertically, controlled co-deposition of dual-component hydrogels can be achieved.

The nozzle was printed using the Formlabs Form 3 desktop stereolithography 3D printer (Formlabs, Chicago, USA). Rigid resin was chosen as the printing material to ensure the structural precision and strength required for nozzle functionality.

#### 2. Nozzle simulation

The fluid behavior within the hydrogels composite forming nozzle was simulated using the COMSOL Multiphysics^®^ software (COMSOL, Stockholm, Sweden). First, the designed nozzle model was imported into the COMSOL software. A laminar flow field within a unidirectional flow was selected. The fluid was set as incompressible. The fluid density was set to 1000 kg/m^3^. Two viscosity values, 220 and 1000 mPa s, were employed to represent high- and low-viscosity hydrogels, respectively. Finally, the velocities at the nozzle inlet were individually set to 0.2, 15, and 30 mm/s to investigate the behavior of the fluid under different flow rates. Once all parameters were configured, the simulation experiments on fluid velocity were conducted.

### C. Preparation of hydrogel

We weigh Alg powder (Macklin, Shanghai, China) and add it to ultrapure water. We stir the mixture with a glass rod for 5 min. The pre-crosslinked Alg hydrogel can be obtained by using a CaCl_2_ solution (Macklin, Shanghai, China). Alternatively, colored Alg hydrogel can be produced by introducing ink dye. Moreover, MP powder (Jiangyin Beiruisen, Wuxi, China) can also be mixed in the Alg hydrogel.

Once the Alg hydrogel is well-stirred, transfer it into a 10 ml syringe and compact the hydrogel using the syringe plunger. We seal the outlet of the syringe with a sealing membrane and place it in a centrifuge at 1200 rpm for 2 min.

### D. Rheological testing

Rheological evaluation was conducted following the ASTM D4473 test method using a Kinexus Ultra+ rheometer (NETZSCH, Bavaria, Germany). The mechanical properties of the hydrogels were assessed based on the storage modulus (G′) and loss modulus (G″), G′ represents elasticity, and G″ represents viscosity.^72^ The frequency sweep was conducted in the range of 0.1–100 rad/s, at a temperature of 25 °C. A strain of 10% was applied using a parallel-plate geometry with a 50 mm diameter and a 0.9 mm gap height. Both crosslinked and non-crosslinked hydrogel samples (3000 *μ*l) were used.

### E. Viscosity measurements

The viscosity profile measurement of each Alg hydrogel was carried out by Kinexus Ultra+ (NETZSCH, Germany). Circular hydrogel samples with a diameter of 25 mm and thickness of 1 mm were prepared. The temperature was maintained at 25 °C. The variation of hydrogel viscosity under a strain of 10% and shear rates ranges from 1 to 35 Hz.

### F. Hydrogel printing

To secure the pre-loaded syringe containing the hydrogels, we use the syringe holder clamp to attach it to the designated position of the portable *in situ* printer. Before starting the printing process, we adjust the printing parameters of the printer and experiment with different settings to determine the best printing outcome. We set the nozzle movement speed to 11.78 mm/s and the hydrogel feed rate to 3 ml/h. To distinguish between the two hydrogels components in multi-component material extrusion, we apply red and blue ink markings. Then, we proceed with hydrogels *in situ* forming using the parameters mentioned earlier. Finally, we use a Nikon-Ti2-D-PD optical microscope (Nikon, Japan) to observe the printing results.

### G. Fiber diameter and spacing measurement

Measuring the diameter of hydrogel fibers was conducted using the ImageJ software. The procedure involved the following steps: 16 positions were chosen on hydrogel fibers, while utilizing a 10× objective lens, in the experimental images. The diameters of the fibers were then recorded at random positions. Each measurement was performed on a single fiber cross section, and the resulting values for fiber diameter were extracted and averaged. The identical approach was applied to measure the spacing between fibers.

### H. Mechanical stiffness

The stiffness of each hydrogel was assessed using an atomic force microscope (AFM, Dimension Edge, Bruker, Germany). To construct AFM cantilever tips, probes (ScanAsyst Air, Bruker, Germany) were attached to tuples silicon cantilevers. These tips were employed to gently apply pressure (0.3 N/m) to the hydrogel surface at a speed of 1.0 *μ*m/s, allowing for the measurement of its stiffness. The obtained data were analyzed and selected using the NanoScope Analysis 1.8 software, and the Young's moduli were determined by fitting the Hertz model to the force curve.

### I. Cell culture

For the experiments, HEK293T cells (293T) were seeded in a culture dish and maintained in a culture incubator. The cells were maintained at a concentration of 5 × 10^4^ cells/ml at 37 °C in a humidified atmosphere of 95% air and 5% CO_2_. The culture medium used was Dulbecco's modified Eagle's medium (DMEM) supplemented with 10% heat-inactivated fetal bovine serum (FBS), 100 U/ml penicillin, and 100 *μ*g/ml streptomycin. When the cells reached 90% confluence, they were treated with trypsin for digestion and then sub-cultured at a ratio of 1:3.

### J. Preparation of cell-loaded hydrogel

After cell culture and sub-culturing, an appropriate amount of cells was obtained. The cells were rinsed by adding phosphate buffer saline (PBS) to the culture dish, followed by the addition of 1 ml of 0.2% trypsin. Once complete cell detachment was observed under a microscope, 5 ml of DMEM growth medium was added to halt the trypsin digestion. Subsequently, the mixture was centrifuged at 4 °C and 1000 rpm for 5 min. After centrifugation, the supernatant was removed. The hydrogel was combined with the cells using a vortex mixer at 500 rpm for 30 s at room temperature. The cell-laden Alg hydrogel had 5 × 10^6^/ml cells.

### K. Cell viability

Cell viabilities were assessed using a live/dead cell assay kit (Solarbio, Beijing, China). Prior to introducing the fluorochromes, cells were washed twice with PBS. The cells were then incubated with calcein-AM staining solution at 37 °C in the dark for 20 min. After removing excess staining solution, the cells were incubated with propidium iodide (PI) staining solution at room temperature in a light-avoiding environment for 5 min. The stained cells were visualized using a Nikon-Ti2-D-PD fluorescence microscope (Nikon, Japan), with live cells emitting green fluorescence and dead cells emitting red fluorescence. The numbers of live and dead cells were quantified separately using the ImageJ software. Cell viability was calculated by dividing the number of live cells by the total number of cells.

### L. Statistical analysis

Data are presented as mean ± standard deviation (SD). Statistical analysis was conducted using GraphPad Prism software (version 9.0; La Jolla, CA, USA). Pairwise comparisons were performed using Student's t-test, while multiple group comparisons were conducted using one-way analysis of variance (ANOVA) followed by Tukey's test. Statistical significance was defined as p < 0.05, p < 0.01, and p < 0.001.

## SUPPLEMENTARY MATERIAL

See the supplementary material for the hydrogel composite printing nozzle and extrusion simulation, the measurement of mechanical stiffness in printing hydrogels, the schematic diagram of the portable *in situ* printer structure, the control system of the portable *in situ* printer, and the schematic diagram of the control system circuit.

## Data Availability

The data that support the findings of this study are available from the corresponding author upon reasonable request.

## NOMENCLATURE

Alg: Alginate
ANOVA: One-way analysis of variance
DMEM: Dulbecco's modified eagle's medium
EthD-1: Ethidium homodimer-1
FBS: Fetal bovine serum
FDM: Fused deposition modeling
G′: Storage modulus
G″: Loss modulus
GelMA: Gelatin methacryloyl
HA: Hyaluronic acid
HAMA: Hyaluronic acid methacrylate
MP: Mussel adhesive protein
MSC: Mesenchymal stem cells
PCB: Printed circuit board
PI: Propidium iodide
PLA: Polylactic acid
SD: Standard deviation
UV: Ultraviolet
3D: Three-dimensional
